# Pharmacodynamics, Network Pharmacology, and Pharmacokinetics of Chinese Medicine Formula 9002A in the Treatment of Alzheimer’s Disease

**DOI:** 10.3389/fphar.2022.849994

**Published:** 2022-04-08

**Authors:** Chunlan Tang, Zhiling Fang, Donghui Chu, Lulu Zhang, Yuqing Tang, Jinyue Zhou, Rui Fang, Jiaming Ying, Fang Wang, Yuping Zhou, Chunshuang Xu, Qinwen Wang

**Affiliations:** ^1^ The Affiliated Hospital of Medical School, Ningbo University, Ningbo, China; ^2^ School of Medicine, Ningbo University, Ningbo, China; ^3^ Key Laboratory of Advanced Mass Spectrometry and Molecular Analysis of Zhejiang Province, Ningbo University, Ningbo, China; ^4^ Department of Translational Neuroscience, State Key Laboratory of Medical Neurobiology and MOE Frontiers Center for Brain Science, Institutes of Brain Science, Fudan University, Shanghai, China

**Keywords:** Chinese medicine formula 9002A, Alzheimer’s disease, UPLC-MS/MS, salidroside, network pharmacology

## Abstract

Alzheimer’s disease (AD) is a common and serious neurodegenerative disease in the elderly; however, the treatment of AD is still lacking of rational drugs. In this paper, the active constituents and targets of the self-developed Chinese medicine Formula 9002A in the treatment of AD were investigated from three aspects: pharmacodynamics based on cell and animal experiments, network pharmacology analysis, and pharmacokinetic analysis. A total of 124 compounds were screened in Formula 9002A, and four constituents including salidroside, gastrodin, niacinamide, and umbelliferone were screened as potential active components for the treatment of AD by network pharmacology. Among them, salidroside and gastrodin showed higher relevance with AD targets, such as ESR1 and AR. The pharmacokinetic study showed that they could be absorbed and identified in plasma; the half-life and mean residence times of salidroside and gastrodin in plasma were nearly increased 2-fold by the administration of Formula 9002A compared with those by the administration of a monomer, indicating the extended action time of active compounds *in vivo*. Formula 9002A exerted the efficacy in the treatment of AD mainly by regulating APP, GSK3β, ESR1, and AR targets based on the anti-β-amyloid protein deposition, anti-oxidation and anti-apoptosis pathways. Two genes enriched in Alzheimer’s disease pathway, APP and GSK3β, were further validated. The experiments also demonstrated that Formula 9002A could downregulate APP and GSK3β protein expression in the model mice brain and improved their cognitive ability. In summary, Formula 9002A has the characteristics of multiple targets and multiple pathways in the treatment of AD, and salidroside and gastrodin might be the main active constituents, which could provide a foundation for further research and application.

## 1 Introduction

COVID-19 is sweeping the world, and many countries are still at risk now. Quarantine measures are considered effective anti-epidemic measures. However, the social isolation of the elderly is considered a “serious public health problem” because they are at the higher risk of cardiovascular disease, autoimmune, neurocognitive, and mental health problems (Armitage and Nellums, 2020). A total of 4,226 case reports in the United States showed that more than 80% of deaths in adult patients occurred over the age of 65 (Shahid et al., 2020). Alzheimer’s disease (AD) is a very common and burdensome neurodegenerative disease in the elderly. Its prevalence and mortality rate are increasing much faster than previous researchers have expected. Between 2000 and 2018, the death toll increased by 146.2%. From the national financial perspective, the total cost of medical, long-term care, and hospice services for dementia patients are detrimental to the development of the country. In addition, Alzheimer’s patients have a higher risk of contracting COVID-19, which is supported by data (Mok et al., 2020), and some studies have explained it from the genetic level (Kuo et al., 2020). *ApoE* e4e4 is a gene known to be associated with AD, which not only affects lipoprotein function and subsequent cardiovascular disease but also reduces the pro-inflammatory/anti-inflammatory phenotype of macrophages, which results in the higher risk of severe COVID-19 (Tudorache et al., 2017). These data demonstrated that much more attention should be paid to the basic health problems of the elderly after the outbreak of COVID-19.

Traditional Chinese Medicine (TCM), a time-honored discipline, has performed well in the prevention and treatment of COVID-19. In fact, the treatment of neurodegenerative diseases of TCM is also widely and historically documented. A 22-year study on the benefits of taking the extracts of *Ginkgo biloba* L*.* on the long-term risk of dementia and death in the elderly showed that patients who were given *Ginkgo biloba* L*.* had a lower mortality rate (Dartigues et al., 2017). Similar results were observed in the study on the effects of *Ginkgo biloba* L*.* on AD symptoms; patients taking *Ginkgo biloba* L*.* showed significant improvement (Savaskan et al., 2018). Our laboratory jointly developed a Chinese Medicine Formula 9002A with many Chinese medicine experts based on their clinic experiences and some references. The preliminary study has shown the good efficacy in the prevention and treatment of AD, which had applied the Chinese patent (Patent number: 201811434083. X). But the detailed efficacy and action mechanism of Formula 9002A have not been carried out. TCM usually acts synergistically according to different characteristics, which is essential for complex AD. Compared with some acetylcholine inhibitors with specific targets, the therapies of multitargets and multipathways of TCM have attracted more attention. In the meantime, another feature of TCM is its complex composition, which sometimes makes it difficult to clarify the specific role of substances and pathways. The combination of network medicine (NM) and Chinese medicine science is exactly in line with the trend. The core idea of NM is that diseases affect not only individual genes through mutations but also many genes and broader pathways (Bedoya-Pilozo et al., 2018). Network pharmacology (NP) is an emerging field of systems biology, which is a perfect combination of the above two. NP can generate complex interaction networks based on target molecules, biological functions, and active compounds to achieve the purpose of explaining mechanisms at the molecular level, which meets the development needs of TCM formulas (Luo et al., 2020). The main procedures of NP were as follows: 1) study its components of TCM, 2) predict the targets and analyze the pathways, and 3) explore the mechanism of TCM and verify through biochemical experiments (Zhou et al., 2020).

Based on the composition complexity of TCM and poor absorption *in vivo*, it is considered that the absorbed components of TCM might be the authentic active substances. In recent years, high-performance liquid chromatography coupled with mass spectrometry (HPLC-MS) has been widely used in the analysis of *in vivo* components of TCM with its high sensitivity and resolution. Shaw et al. (2012) used HPLC-MS/MS to detect nine components in Bu-Yang-Huan-Wu-Tang and studied their pharmacokinetics. Nie et al. (2020) applied the NP analysis method to study the pathological mechanism of Chaihu Shugan powder in the treatment of non-alcoholic fatty liver disease and detected five components in the powder by HPLC-MS. There are many similar studies on the application of HPLC-MS/MS to the active ingredients, metabolic kinetics, and metabolomics of TCM. It can be seen that HPLC-MS/MS has become the mainstay technology of TCM analysis. In addition, research also showed that there were some mutual influences between different ingredients; a specific herbal combination ratio is critical for maximum synergetic action (Su et al., 2016).

This article integrated the pharmacodynamics and network pharmacology to evaluate and analyze the efficacy, potential active constituents, and action mechanism of Formula 9002A. Furthermore, the absorbed components *in vivo* and some pharmacokinetic parameters of Formula 9002A were investigated, and the screening targets were verified by biochemical experiments.

## 2 Materials and Methods

### 2.1 Experimental Cells and Animals

SH-SY5Y cells were purchased from Shanghai Cell Bank, Chinese Academy of Sciences. All ICR mice (mean 30 ± 5 g) for pharmacodynamics and SD rats (200 ± 20 g) for pharmacokinetic study were purchased from Zhejiang Laboratory Animal Center. They were housed in a temperature- and humidity-controlled room with a 12-h light/dark cycle, with free access to food and water. All experimental protocols on animals were in accordance with the guidelines of the Committee on the Care and Use of Laboratory Animals of China and approved by Animal Ethics and Welfare Committee of Ningbo University.

### 2.2 Reagents

Aβ_1-42_ (Shanghai GL Bio-Chem Ltd.) and hexafluoroisopropanol (HFIP, Shanghai Aladdin Bio-Chem Technology Co., Ltd.) were used to synthesize Aβ oligomers. The formula consists of *Gastrodia elata Blume*, *Curcuma longa L.*, *Punica granatum L.*, *Morus alba Linn.*, *Lycium barbarum* L, *Ginkgo biloba*, *Rhodiola rosea,* and *Alpinia oxyphylla Miq.* The herbs were purchased from Beijing Tongrentang Pharmaceutical Co. Ltd. Dulbecco’s modified Eagle’s medium (DMEM) was purchased from Hyclone (United States). Penicillin, streptomycin, and trypsin were purchased from Beyotime Biotechnology (China). Phosphate buffered saline (PBS) was purchased from Solarbio Life Sciences (China), and 3-(4,5-dimethyl-2-thiazolyl)- 2,5-diphenyl-2-H-tetrazolium bromide (MTT) was bought from Sigma (United States). Amyloid beta A4 rabbit antibody (Catalog No: A17911) and GSK3β rabbit antibody (Catalog No: A11731) were purchased from Wuhan ABclonal Biotech Co., Ltd. The standard products of salidroside, umbelliferone, gastrodin, and nicotinamide were purchased from Yuanye Biotechnology Co., Ltd. (China). Acetonitrile and methanol were supplied by Merck KGaA (Darmstadt, Germany). Formic acid of chromatographic grade was purchased from Aladdin (Shanghai, China). Ultrapure water was prepared by a water purification system (Milli-Q Integral 5). All other chemicals were of analytical grade.

### 2.3 Preparation of Formula 9002A

According to the fixed proportion in the patent application, the eight herbs including *Gastrodia elata Blume* (20%), *Curcuma longa L.* (20%), *Punica granatum L.* (10%), *Morus alba Linn.* (10%), *Lycium barbarum* L. (10%), *Ginkgo biloba* (5%), *Rhodiola rosea* (10%), and *Alpinia oxyphylla Miq.* (5%) were mixed and extracted by pure water at a ratio of 1:10; the filtrate was collected after 2-h decoction and concentrated in a rotary evaporator, and the extracted rate was 0.47 g/g raw medical material.

### 2.4 Cell Experiments

#### 2.4.1 Preparation of Aβ_1-42_ Oligomer

Synthesized Aβ_1-42_ powder (5 mg) was dissolved in 2-ml HFIP to form 2.5 mg/ml Aβ_1-42_ monomer, maintained at room temperature for 20 min. Then it was dispensed into 1.5-ml EP tubes with 100 μl per tube and stored at −20°C. A tube of Aβ_1-42_ monomer was added with 900 μl of ultrapure water then maintained at room temperature for 10 min. HFIP was removed with high purity nitrogen until the volume was at 700–750 μl. Furthermore, the Aβ_1-42_ solution was oscillated in a magnetic stirrer at room temperature for 24 h to form Aβ_1-42_ oligomers and centrifuged at 4°C with 15,000 g for 15 min, and the soluble Aβ_1-42_ oligomers in the supernatant were collected.

#### 2.4.2 Cell Culture

SH-SY5Y cells were cultured in DMEM supplemented with 10% FBS, 100 μg/ml penicillin, and 100 μg/ml streptomycin and maintained at 37°C and 5% CO_2_ in a humidified incubator. When cells reached 80% confluency, they were digested with 0.25% trypsin and were then collected, resuspended, and cultured.

#### 2.4.3 Cell Viability Assay

Cell viability was detected by MTT assay. Formula 9002A was diluted to 0.5 mg/ml with DMSO. SH-SY5Y cells were planted on 96-well plates at a density of 7 × 10^3^ cells/well in a humidified atmosphere of 5% CO_2_ at 37°C. After 24 h, the cells were infected with 1 μM Aβ_1-42_. DMEM of 10 μl Aβ_1-42_ and 190 μl was added to each well; some wells were reserved for the control group, and 200 μl Formula 9002A (0.5 mg/ml) was added to the well after 2 h as the treatment group. After incubation for 24 h, cell cultures were replaced with DMSO (150 μl/well). After shaking the plate at 37°C for 10 min, the absorbance was measured with a tablet reader at 570 nm. The experiment was divided into three replicates, and the survival rate was calculated as the following equation:

$$\text{Viability rate}\left(\text{\%}\right)=\left[\left({\text{OD}}_{\text{Sample group }}-{\text{ OD}}_{\text{Blank}}\right)/\left({\text{OD}}_{\text{Control }}-{\text{ OD}}_{\text{Blank}}\right)\right]\text{×}100\text{\%}$$

### 2.5 Animal Experiment for Pharmacodynamics Study

#### 2.5.1 Modeling and Administration

ICR mice were divided into three groups: control group, Aβ model group, and treatment group with six mice in each group. The control and Aβ model groups were kept normally without additional food. The treatment and Aβ model groups received surgical embedding on the 18th day of rearing, and Aβ_1-42_ polymer was injected through the brain on the 24th–26th day. Additionally, the mice in the treatment group were given intragastric administration of Formula 9002A at a dose of 3 g/kg/day for 36 consecutive days. The behavior tests were carried out from the 27th to the 36th day, then the brain tissue samples of each group of mice were taken and placed at −80°C until use.

#### 2.5.2 Behavior Test

Open field test (OFT), new object recognition test (NOR), and Morris water maze (MWM) were conducted. The detailed processes of OFT, NOR, and MWM are provided in Supplementary Material S1. The data are expressed as means ± SD. Statistical significance was determined by one-way ANOVA and Tukey’s or Dunnett’s test for *post hoc* multiple comparison. The mean escape latency was analyzed using two-way repeated-measures ANOVA followed by LSD *post hoc* test. *p* < 0.05 was considered as statistically significant.

#### 2.5.3 Western Blot Experiment

After homogenizing the brain tissues of mice, the protein was extracted with NP40 lysate (protein phosphatase inhibitor mixture was added), and the protein concentration was determined by the BCA protein concentration analysis kit. Proteins (30 µg) were loaded in 10% SDS polyacrylamide gel for electrophoresis. The electrophoretic products were transferred to a PVDF membrane, which were further added with 1:1,000 diluted primary antibody and incubated at 4°C overnight. After rinsing with TBST (Tris Buffered Saline Tween), the membranes were incubated with the secondary antibody at room temperature for 1 h (Zou et al., 2019). ImageJ 1.53 A was used to calculate the gray value, and comparisons among the three groups and internal reference were carried out.

### 2.6 Network Pharmacology

#### 2.6.1 Compound Screening

The compounds’ information of Formula 9002A was obtained from the TCMSP database. The active compounds were filtered by integrating oral bioavailability (OB ≥ 30%), drug-likeness (DL ≥ 0.18), and blood-brain barrier permeability (BBB≥0.3). In order to avoid missing ingredients that have been proven to treat AD but do not meet the screening criteria, relevant literatures were searched for and active substances were added for further research.

#### 2.6.2 Target Prediction

The drug–target interaction prediction was performed by the TargetNet server, which uses several strategies to strictly evaluate and verify the possibility of target interactions with human proteins (Yao et al., 2016). The 3D structures of active compounds were imported into the TargetNet server separately, and proteins with a score greater than 0.5 were selected as potential target proteins. Furthermore, the Uniprot database was used to convert the “protein names” of the targets into “gene names” uniformly for subsequent analysis. In the meantime, genes related to AD were searched in GeneCards and TTD, which are two databases that summarize genes based on diseases. Finally, the relevant targets of Formula 9002A predicted by the TargetNet server were mapped to the AD-related targets, and the intersection genes in the two collections were considered to be the effective targets of the active ingredients of Formula 9002A in the treatment of AD.

#### 2.6.3 Pathway Enrichment Analysis

Kyoto Encyclopedia of Genes and Genomes (KEGG) pathways and Gene Ontology (GO) enrichment analysis on the target group of Formula 9002A were conducted through the DAVID database. *Homo sapiens* was set for operation. The top 20 GO items and metabolic pathways of the active compounds of Formula 9002A related to AD were screened out.

#### 2.6.4 Network Construction

Two visualized networks were constructed by the STRING database and Cytoscape 3.7.1: 1) protein–protein interaction (PPI) of intersection genes from predicted targets of Formula 9002A and AD-related targets and 2) compound-target-pathway (C-T-P) network.

### 2.7 Pharmacokinetic Analysis of Formula 9002 A

#### 2.7.1 Sample Preparation

For *in vivo* absorbed constituent analysis, the rat blood (*n* = 3) was collected into vacuum blood vessels containing heparin sodium from the abdominal aorta of rats after 1 h of administration of Formula 9002A (2.1 g/kg), and the blood of the control group was also collected. For pharmacokinetic study, serial blood samples (100 μl) were collected into heparin-containing tubes from the ophthalmic artery plexus of rats using capillary tubes at 0.083, 0.25, 0.5, 0.75, 1, 2, 4, 6, 8, 12, and 24 h after administration of Formula 9002A at a dose of 2.1 g/kg. All blood samples were promptly centrifuged at 15,000 g for 15 min, the plasma section was separated, and iced-cold methanol in a ratio of 1:3 (v/v) was added to precipitate the protein. The supernatant were obtained and concentrated, then dissolved with 50% methanol–water at 4°C for LC-MS/MS analysis.

#### 2.7.2 UPLC-QTof-MS Analysis

Waters Xevo G2-XS QTof-MS and Masslynx software were used to acquire LC-MS/MS raw data. Chromatographic separation on the Waters Acquity UPLC HSS T3 column was performed on the samples. The mobile phases consisted of (A) 0.1% formic acid in water and (B) acetonitrile. The UPLC elution condition was optimized as follows: linear gradient from 3% to 40% B (0–5.0 min), 40–95% B (5.0–8.0 min), maintaining 95% B (8.0–10.5 min), 95–3% B (10.5–11.0 min), and maintaining 3% B (11.0–13.0 min). The flow rate was set at 0.3 ml/min. The temperatures of the column and the auto sampler were maintained at 40 and 4°C, respectively. The injection volume was 5 μl.

MS conditions: positive and negative ion electrospray modes were both used. Capillary voltages were set at 3 kV for the positive mode and 2.5 kV for the negative mode. Source temperature was set as 100 °C. Nitrogen was used as the nebulizer and auxiliary gas; flow rates and temperature of desolvation gas were 1,000 L/h and 400°C, respectively. The range of data acquisition was from 50 to 1,000 m/z, and the scan time was set to 0.2 s. MS^E^ analysis was performed with 6 eV for the low collision energy scan and 35 eV for the high collision energy scan.

#### 2.7.3 Pharmacokinetic Study

The absorbed components were identified by the exact molecular mass, molecular formula, and MS^2^ fragment data compared with the standard substances and literature data. The DAS 3.3.0 software was used for calculating the terminal elimination half-life (t_1/2_) and mean residence time (MRT).

## 3 Results

### 3.1 Formula 9002A Improved Cell Viability of SH-SY5Y Induced by Aβ Oligomers

**FIGURE 1:**
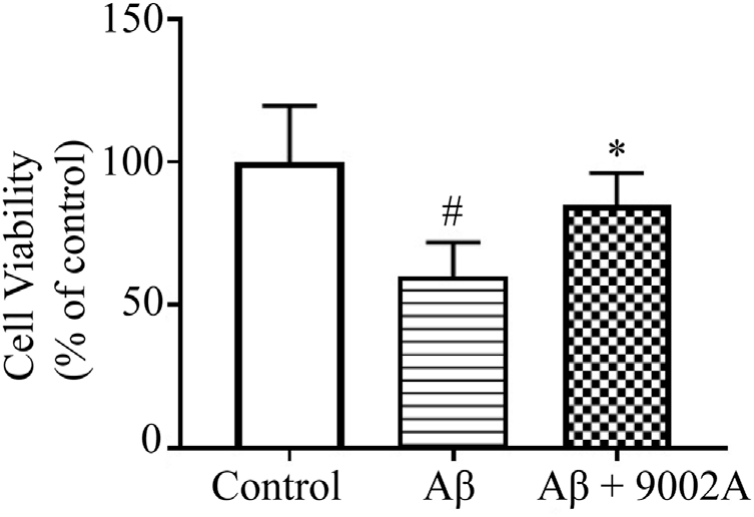
Cell viability of SH-SY5Y cells in the control group, the Aβ model group, and the treatment group. ^**#**^ *p* < 0.05 vs. control group, *p < 0.05 vs. Aβ model group (one-way ANOVA and Dunnett-*t* test).

Accumulation of extracellular amyloid plaque is considered to be a pathological feature of AD, and soluble Aβ oligomers may contribute to cognitive impairment. Therefore, the neuroprotective effects of Formula 9002A against Aβ oligomers-induced neuronal death in SH-SY5Y cells were investigated. The results showed that Aβ oligomers could significantly reduce the cell viability of SH-SY5Y cells, and the treatment group could improve the survival rate of SH-SY5Y cells compared with the Aβ model group (*p* < 0.05, Figure 1), suggesting that Formula 9002A has a neuroprotective effect on SH-SY5Y cells induced by the Aβ oligomer.

### 3.2 Formula 9002A Alleviated the Learning and Memory Impairment Induced by Aβ Oligomers

**FIGURE 2:**
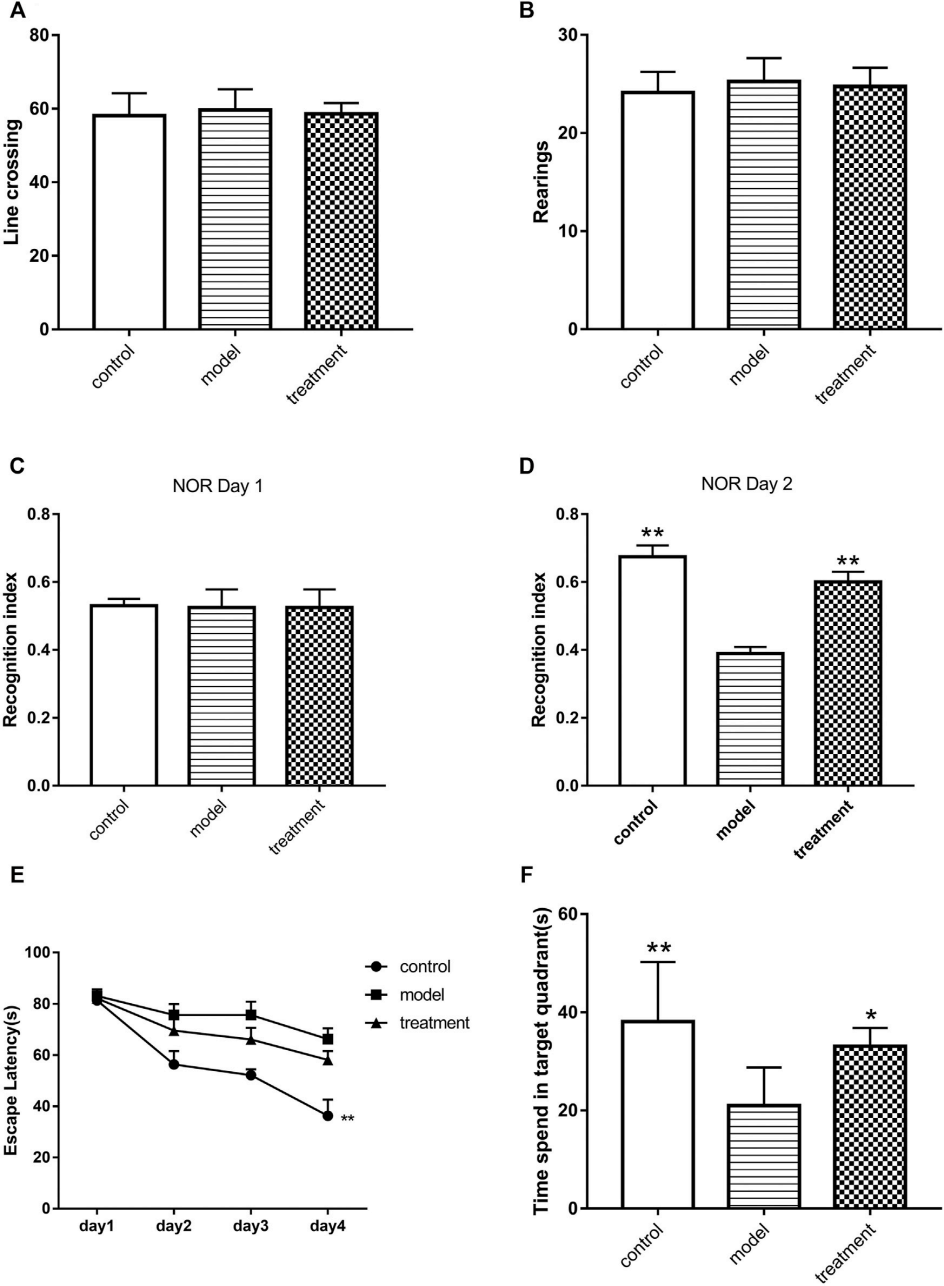
The results of animal behavior experiments. **(A–B)** The times of mice for crossing lines **(A)** and rearing **(B)** in the OFT; in the **(C)** 1st day and **(D)** 2nd day of NOR, the recognition index of mice for two identical and new objects; **(E)** during the 4-day training period of MWM, the time of each group of mice to find the platform; **(F)** after the platform was removed on the 5th day of MWM, the time of mice to spend in the target quadrant. Data represent the mean ± SEM (*n* = 6); ^*^ *p* < 0.05 and ^**^ *p* < 0.01 *versus* Aβ oligomer model group (one-way ANOVA and Dunnett-*t* test).

The exercise ability of mice in each group was measured by OFT. The results showed that injecting the Aβ oligomer into the hippocampus did not affect the exercise ability of mice by counting the times of standing and crossing the line (*p* > 0.05, Figures 2A,B). Furthermore, the efficacy of Formula 9002A on Aβ oligomer-induced cognitive dysfunction in mice was evaluated by NOR test based on the time spent exploring two identical objects. The results showed that the recognition index of two identical objects in each group had no statistical difference on the first day (*p* > 0.05, Figure 2C), whereas on the second day of NOR test, the recognition index of the Aβ oligomer model group was significantly lower than that of the control group, and Formula 9002A could significantly reverse the reduction in the recognition index induced by the Aβ oligomer (*p* < 0.01, Figure 2D). MWM is a classic method used to assess learning and memory in animals. On day 4 of the training period, the mean time required for the control mice to find the platform was reduced to 36 s, while longer time (66 s) was needed for the Aβ oligomer model mice to find the platform (*p* < 0.01, Figure 2E). For the treatment group, the mean time (58 s) was less than that in the Aβ oligomer group, but there was no statistical difference. Finally, the platform was removed on day 5 of MWM. The exploration time of mice in each group was recorded in the target quadrant shown on the platform. The results showed that mice in the control group spent about 38 s in the target quadrant, and the shortest exploration time of mice in the target quadrant was 21 s. The exploration time of mice in the Formula 9002A treatment group increased to 31 s in the target quadrant (*p* < 0.05, Figure 2F). These results indicated that the mice treated with the Aβ oligomer had severe learning and memory impairment, while Formula 9002A can alleviate the learning and memory impairment induced by the Aβ oligomer.

### 3.3 Network Pharmacology

#### 3.3.1 124 Compounds and 101 Targets Were Screened Out

A total of 124 active compounds of Formula 9002A (Supplementary Material S2) were obtained. The 3D structures of all compounds were downloaded from PubMed and imported into the TargetNet (http://targetnet.scbdd.com) for target prediction. The structures of some compounds are invalid and can be ignored. The proteins were chosen as potential targets with a score greater than 0.5. A total of 110 potential target proteins were listed and further converted into “gene names.” Meanwhile, gene entries related to AD are collected from CTD and GeneCards databases. As a result, 22,150 and 6,634 protein-coding gene entries were obtained from these two databases. Among the 110 hypothetical targets, 101 can be matched with AD-related targets in the database (Supplementary Material S3). Many clinically validated targets related to AD were caught, including APP, neuronal acetylcholine receptor subunit α-7 (CHRNA7), acetylcholinesterase (AChE), GSK3β, prostaglandin GH synthase 1, 2 (PTGS1 and PTGS2), and so on.

#### 3.3.2 GO Enrichment and KEGG Pathways Analysis

**FIGURE 3:**
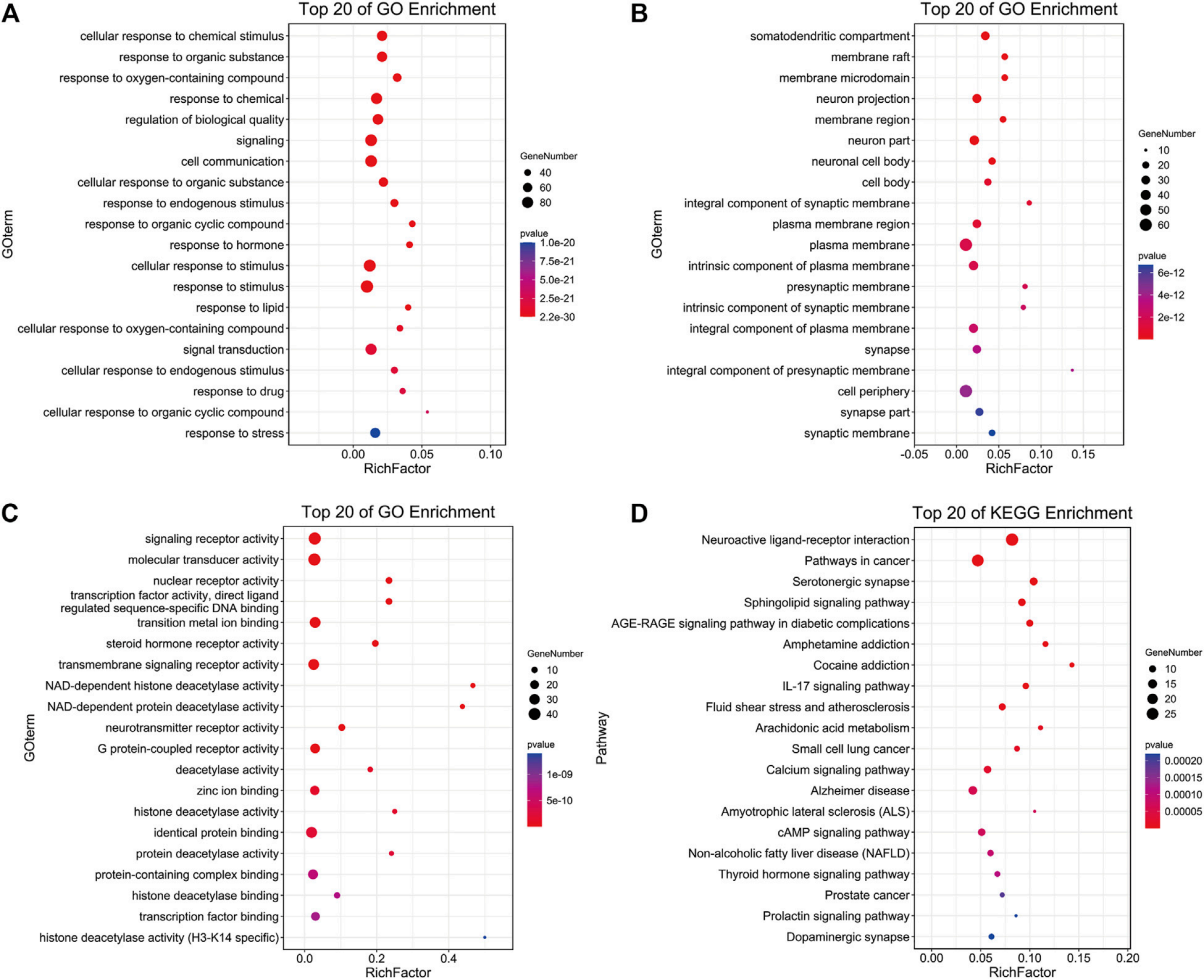
The enrichment results of CC **(A)**, BP **(B)**, MF **(C),** and KEGG pathway **(D)** of Formula 9002A predicted targets.

The enrichment analysis of 101 potential target genes was carried out at the DAVID database. The results showed that the biological process (BP) of the target genes mainly involves the cell response to different types of external stimuli (chemical stimulus, organic substance, hormone, drug, and stress) and signal transduction. The cellular component (CC) is mainly concentrated in the plasma membrane, neuron activity, and synapse. Molecular function (MF) is related to various biological enzymes and their receptors (steroid hormone, NAD-dependent histone deacetylase, neurotransmitter, and G protein-coupled receptor). The top 20 items of BP, CC, and MF were displayed in bubble charts. Additionally, 40 KEGG metabolic pathways with *p* values less than 0.01 were obtained, and the top 20 are shown in Figure 3. Among these pathways, neuroactive ligand–receptor interaction, serotonergic synapse, calcium signaling pathway, and Alzheimer’s disease pathway deserve our attention, which are all closely related to neurodegenerative disease.

#### 3.3.3 C-T-P Network and PPI Construction

**FIGURE 4:**
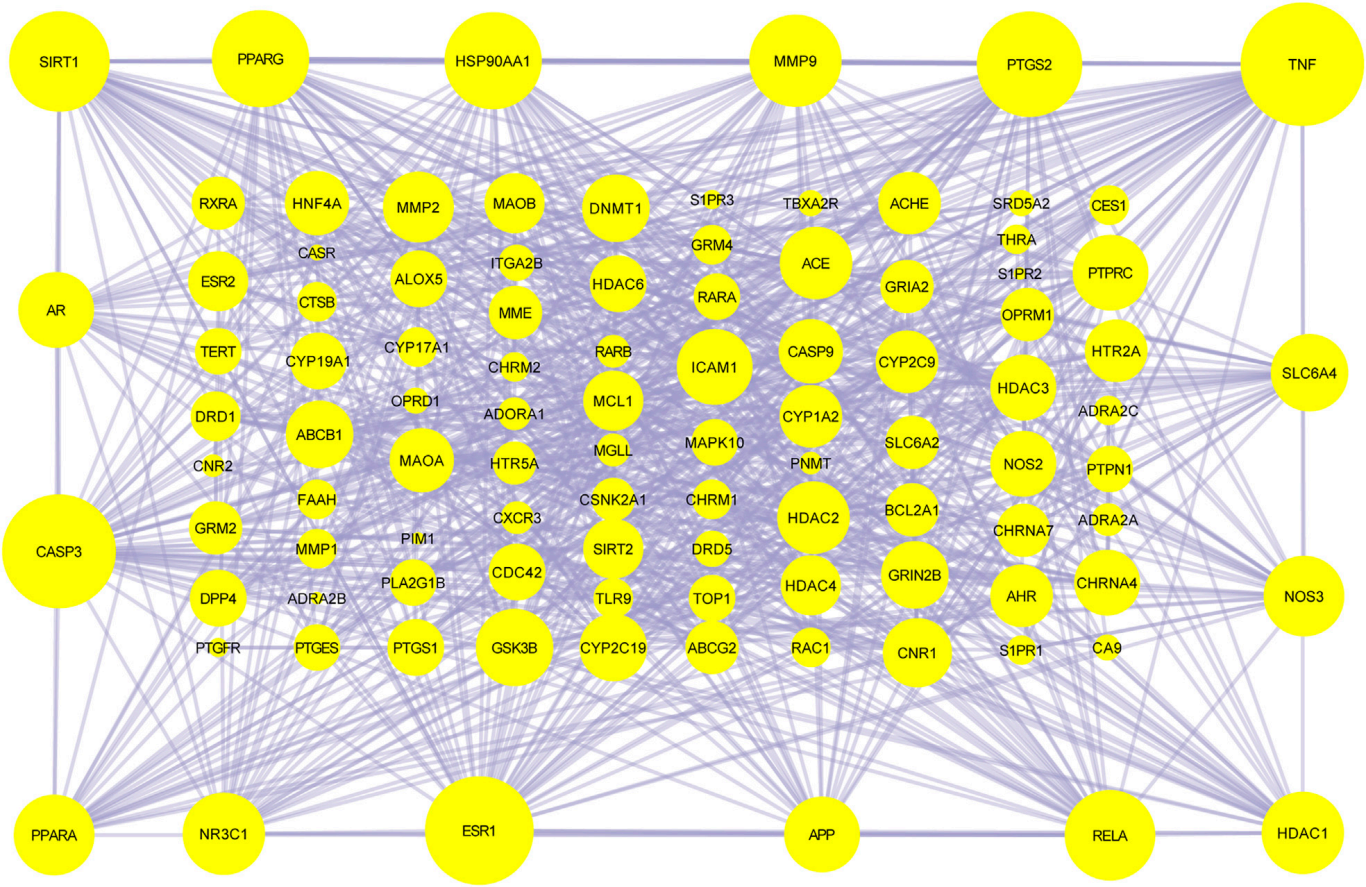
PPI of 101 targets of Formula 9002A associated with Alzheimer’s disease.

**FIGURE 5:**
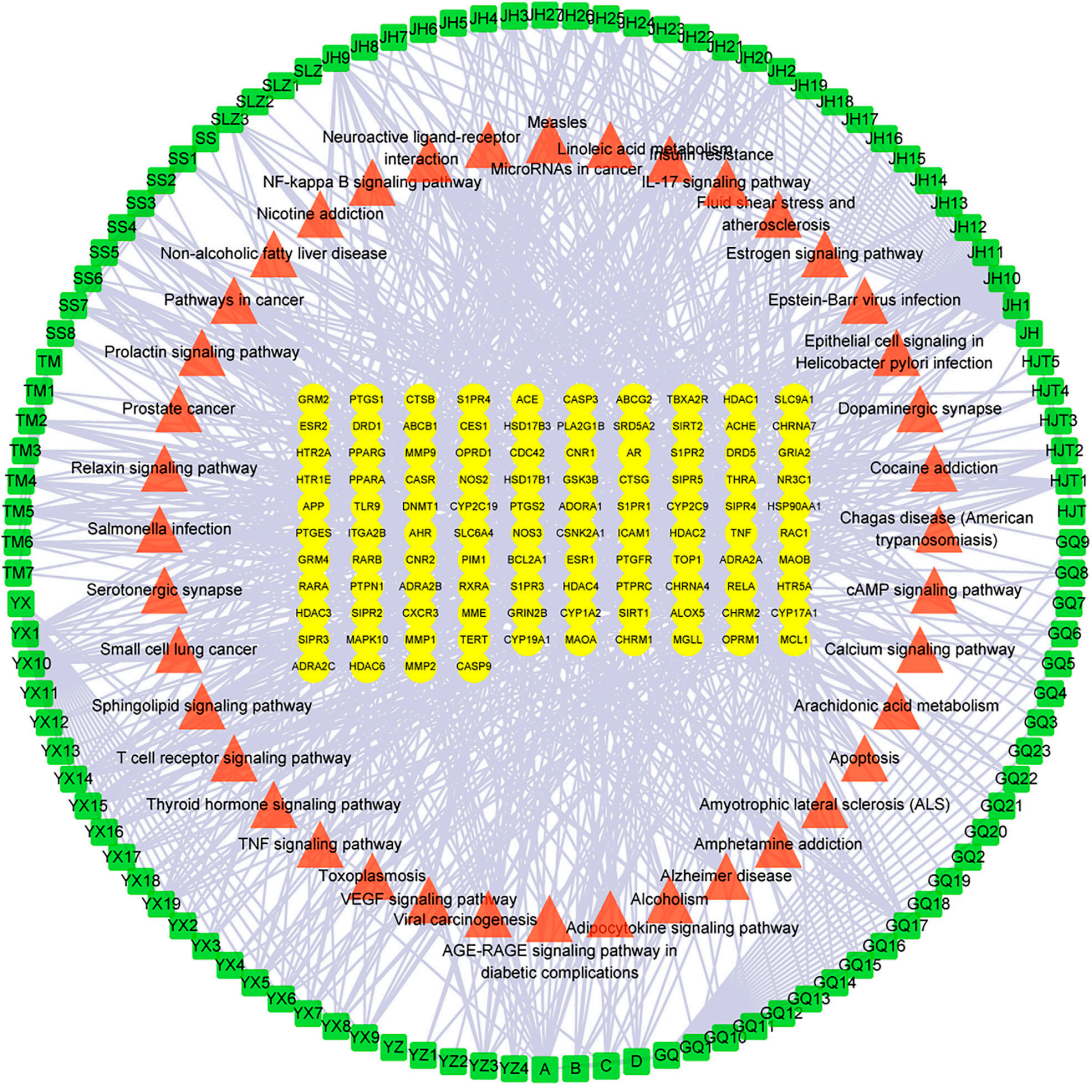
The C-T-P network of Formula 9002A. The outermost green square represents the active component, the red triangle for the pathway, and the yellow oval in the middle for the core targets.

The 101 proteins related to AD were imported into STRING 11.0, and the PPI of the target proteins was constructed with the medium confidence equal to 0.4 as the screening criterion. A diagram of 774 edges was obtained exceeding the expected 236 edges, which indicated that these proteins were biologically linked, as shown in Figure 4. The higher the enrichment, the larger the circle representing the gene. The C-T-P network was completed with 252 nodes and 1,030 edges (Figure 5). The two networks help clarify which components, targets, and pathways are more concentrated.

According to the results, the common component mandenol in *Lycium barbarum* L. and *Ginkgo biloba* has the most correlation (20 edges), followed by turmeronol B and β-pinene in *Curcuma longa L*. with 15 edges as well as benzoic acid in *Morus alba Linn.* In fact, there have been many studies on *Ginkgo biloba* and *Curcuma longa L*. treating AD (Witkin and Li, 2013; Singh et al., 2019). Their extracts can be used for therapeutic purposes through antioxidant, anti-inflammatory, and regulation of cell death. The targets of mandenol include PPARG, CNR1, ALOX5, HDAC3, MGLL, PTGS1, CNR2, S1PR3, TBXA2R, and THRA. The targets of turmeronol B include THRA, CASP3, RELA, RXRA, DNMT1, CASP9, BCL2A1, RARB, and SIRT2. The targets of β-pinene include SLC6A4, NOS3, ADRA2C, ADRA2A, ADRA2B, HTR5A, NR3C1, CHRM2, CHRNA4, DRD1, CYP19A1, GRM2, CNR2, CXCR3, and CYP17A1. The targets of benzoic acid include RELA, HDAC3, MMP9, and HDAC4. Among all the targets involved, RELA has the highest correlation (55 edges); CYP17A1 (35 edges) and CASP9 (14 edges) also have great correlation. In addition, the results of targets have showed that several other genes have a high degree of enrichment. ESR1 has the most directed edges (58 edges), followed by AR (42 edges), PTGS2 (27 edges), MAPK10 (23 edges), ESR2 (22 edges), APP (21 edges), and TNF and NOS3 (both 19 edges). These data indicated that they were probably the action targets of Formula 9002A. ESR1 and ESR2 are classic nuclear estrogens that can coordinate the metabolism of the brain and the body, so that the peripheral metabolic state can indicate the bioenergy state of the brain. Adaptive or inadaptable organisms that compensate for loss of estrogen may determine the risk of late-onset AD (Rettberg et al., 2014). The expression of specific estrogen spliceosomes in the hippocampus increases with age, and several estrogen polymorphisms increase the risk of AD, especially in women and when associated with the *ApoE* e4e4 allele (Ryan et al., 2014). AR is a nuclear steroid hormone receptor and transcription factor, which can be found in both reproductive and nonreproductive tissues of the human body (Christiansen et al., 2020). It regulates gene transcription by binding to specific androgen response elements in DNA, and many evidences support the important role of androgen and AR in the hippocampal function (Raber, 2008). Both estrogen and androgen can promote synaptic plasticity and are powerful modulators of the neuron survival rate, protecting neurons against a range of toxic insults including those implicated in AD (Barron and Pike, 2012). CYP17A1 is a very important enzyme in humans that catalyzes the formation of all endogenous androgens (Porubek, 2013). RELA is a member of the NF-κB family, which is an important transcription factor regulating cellular response and involves several metabolic pathways. The phosphorylated NF-κB subunit is considered to play a role in the course of AD (Yamaguchi et al., 2019). Additionally, the topological analysis data of the C-T-P network in the pathway are consistent with the data of DAVID, and the most relevant pathway is the neuroactive ligand–receptor interaction and Alzheimer disease pathway.

### 3.4 Formula 9002A Reduced the Accumulation of APP and GSK3β

**FIGURE 6:**
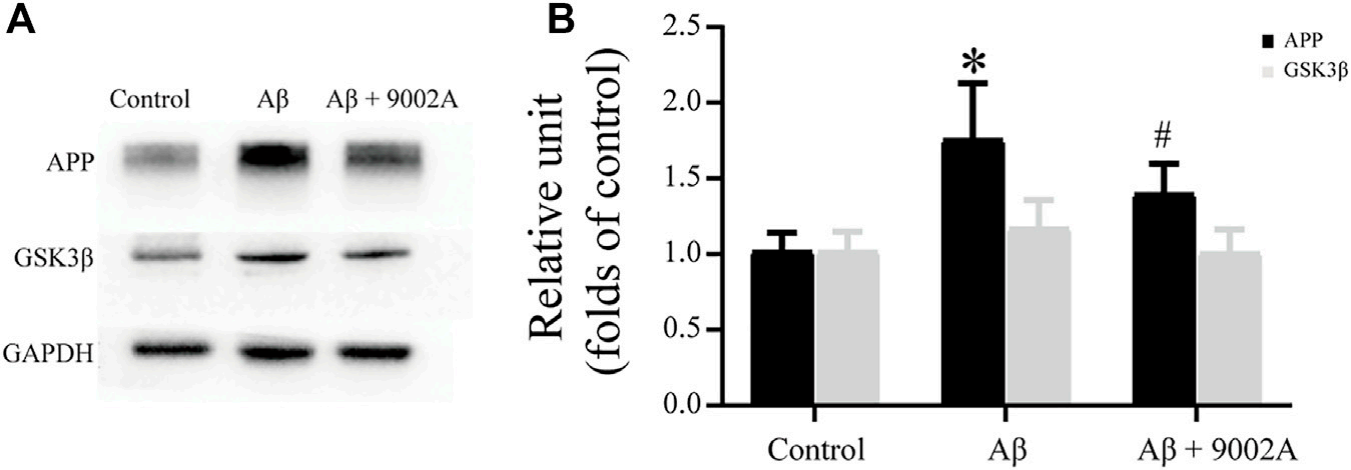
The expression of APP and GSK3β in the mouse brain of the control group, the Aβ model group, and the treatment group.

According to the higher relevant Alzheimer disease pathway and genes from network pharmacology results, two targets including amyloid beta precursor protein (APP) and glycogen synthase stimulate 3β (GSK3β) were further validated based on western blot experiments. As shown in Figure 6, the expressions of APP and GSK3β in the model group were significantly increased compared with those in the control group (*p* < 0.05). Moreover, the APP expression level in the treatment group significantly decreased compared with that in the model group (*p* < 0.05); the expression of GSK3β in the treatment group had a downward trend, but it was not statistically significant, which indicated that Formula 9002A could effectively reduce the accumulation of APP and GSK3β and delay the development of AD.

### 3.5 Pharmacokinetic Study

#### 3.5.1 Identification of Four Components of Formula 9002A *in Vivo*

**FIGURE 7:**
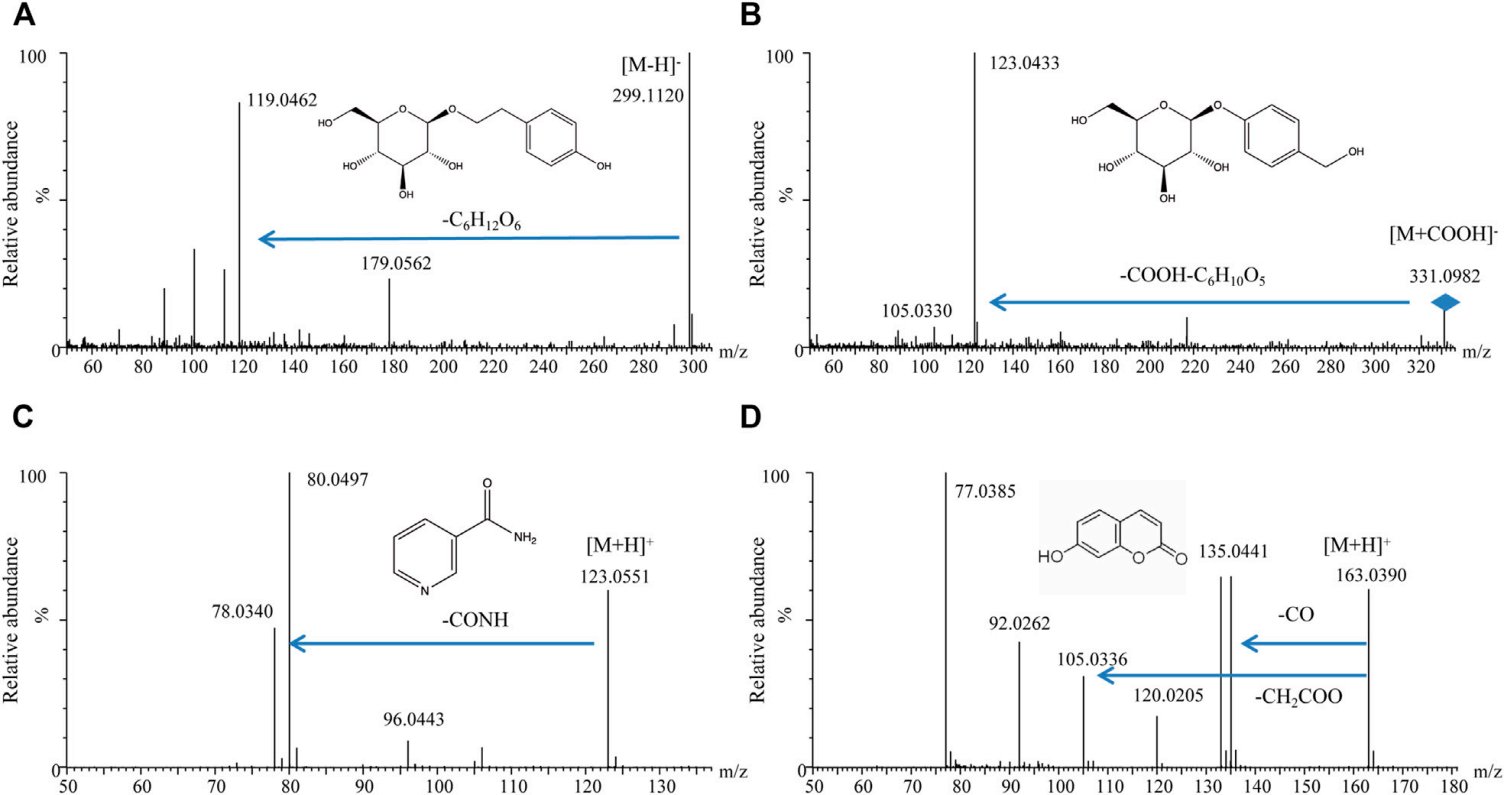
MS^2^ spectrograms of four compounds in rat plasma. **(A)** Salidroside; **(B)** gastrodin; **(C)** nicotinamide; **(D)** umbelliferone.

It is well known that only the components that could be absorbed *in vivo* and showed certain exposed quantity might be considered as the potential active substances. Therefore, the absorbed components of Formula 9002A in plasma were detected by UPLC-MS/MS. Four components, including salidroside, gastrodin, niacinamide, and umbelliferone, were detected and identified by the exact molecular mass, molecular formula, and MS^2^ fragment data compared with the standard substances and literature data; the detail spectrum of these four components is shown in Figure 7.

#### 3.5.2 Pharmacokinetic Study

Among the detected four components, niacinamide and umbrafolactone could only be detected at several discontinuous time points, so no further pharmacokinetic studies were carried out. The pharmacokinetic parameters of salidroside and gastrodin were further investigated. Two parameters of t_1/2_ and MRT of salidroside and gastrodin in rat plasma were calculated. The results showed that the t_1/2_ of salidroside and gastrodin were 2.117 and 2.579 h, and the MRT of salidroside and gastrodin were 2.905 and 3.567 h, respectively.

## 4 Discussions

The treatment of AD is still lacking of rational drug as far as now. Some clinical drugs approved by the Food and Drug Administration (FDA) in the treatment of AD, such as tacrine, donepezil, rivastigmine, and galantamine, showed certain treatment functions, but side effects including nausea, diarrhea, insomnia, and slow rhythm of the heart cannot be ignored (Fang et al., 2020). TCM has been proven to be an effective therapy for alleviating complex diseases in a multitarget/multicomponent manner. In addition, TCM has been used for thousands of years in the treatment of neurological diseases. Formula 9002A was our self-developed Chinese prescription based on the experience of many Chinese medical specialists, and this prescription has been applied for a Chinese patent. In this present study, the pharmacodynamics, network pharmacology, and pharmacokinetic studies were carried out to clarify the neuroprotective effect and potential action mechanism of Formula 9002A.

Cell experiments confirmed that Formula 9002A could significantly improve the survival rate of SH-SY5Y cells induced by the Aβ oligomer. Animal behavior experiments further confirmed that Formula 9002A significantly improved Aβ oligomer-induced cognitive impairment in mice, which both indicated that Formula 9002A exerted neuroprotective effect. Furthermore, the network pharmacology of Formula 9002A was investigated to clarify action mechanism and potential active substances.

The ingredients and targets of Formula 9002A were collected through the integrated pharmacology research platform of TCM. It can be found that one compound may act on multiple targets, and several compounds can act on the same target. The effects are closely relevant, and the drugs complement each other. The analysis of gene function and pathways showed that the intervention effect of Formula 9002A on AD mainly involves β-amyloid protein aggregation, cell apoptosis, inflammation, signal transduction, and so on. The key targets including ESR1, RELA, CYP17A1, APP, TNF, AR, etc. were screened out through constructing a C-T-P interaction network. The appearance of ESR1 and ESR2 in the enrichment results was quite interesting. Prior treatments for AD have focused on the depolymerization of amyloid and the regulation of some neurotransmitters, but the results of the present study suggested that hormones may also play an indispensable role in the course of AD. Although abnormal estrogen levels in AD patients have been confirmed in the literature through clinical samples, the specific mechanism needs to be further verified. In the meantime, regulation of hormone levels can be associated with the characteristics of TCM. TCM, as decoction or other forms of medicine, takes a long time to become effective and hormone levels slowly improved. Therefore, TCM has a potential for treating chronic diseases such as AD.

**FIGURE 8:**
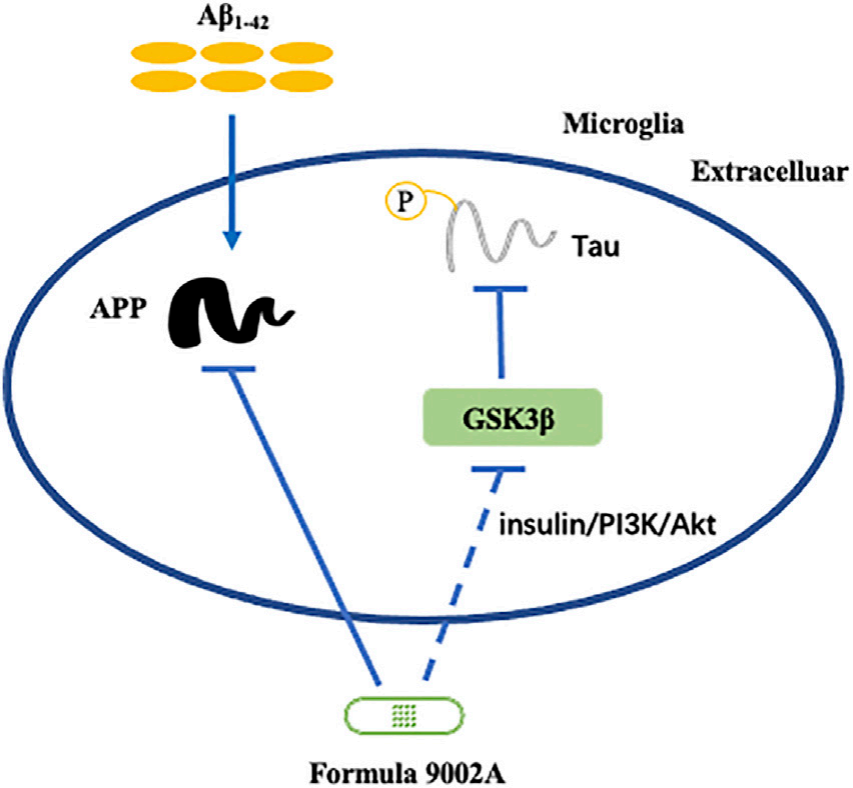
The mechanism of Formula 9002A aimed at APP and GSK3β for AD.

According to our western blot experiment of the brain tissue of mice, it was found that Formula 9002A could significantly downregulate APP expression and also affect GSK3β expression in the mice brain induced by Aβ_1-42_. These results were in consistent with that of network pharmacology. Abnormal accumulation of Aβ is one of the most significant clinical pathological features of AD, which is the result of overexpression of APP. Tau phosphorylation is also a major pathological feature of AD, and GSK3β is one of the important kinases for tau phosphorylation. GSK3β is an important serine/threonine (Ser/Thr) protein kinase and is involved in a variety of biological processes, such as glycogen metabolism, cell proliferation, stem cell renewal, apoptosis and development, etc. (Silva-Palacios et al., 2018). GSK3β is also involved in the insulin/phosphoinositide 3-kinase/protein kinase B (insulin/PI3K/Akt) pathway, whose dysfunction can lead to the hyperphosphorylation of tau in the brain of AD (Zhang et al., 2018). Therefore, Formula 9002A could regulate the expression of APP and GSK3β to reduce the accumulation of Aβ and hyperphosphorylation of tau in the brain for neuroprotective effect. Their mechanism of action is shown in Figure 8.

According to the results of network pharmacology, it was also found out that mandenol, turmeronol B, β-pinene, salidroside, rhodionin, isorhamnetin, beta-carotene, and biflavone showed high relevance with the main targets of AD. These substances were considered as the potential active ingredient for neuroprotective effect. It is known that chemical constituents in Chinese medicine prescription are quite complex; many experts declared that only the absorbed constituents with certain exposure might get to the target position and showed the active action. Therefore, the absorbed components of Formula 9002A in blood were detected. Four compounds including salidroside, gastrodin, niacinamide, and umbelliferon were detected and identified in the administered rat plasma. Nrf2 activated by salidroside could bind to antioxidant response elements (AREs) such as heme oxygenase-1 (HO-1), SOD, and GSH-Px and could be upregulated to mediate neuroprotection and antioxidative stress in cerebral ischemia (Jin et al., 2019). In addition, salidroside reversed the decrease in tight junction proteins (such as claudin-5 and occludin) by inhibiting the activation of MMP-9 (a member of MMPs), which proved the improvement of BBB damage in experimental stroke rat models (Gibson et al., 2014). Gastrodin could upregulate the expression of BDNF to play a neuroprotective effect by regulating the cAMP/PKA/CREB signaling pathway (Ma et al., 2020). Hu et al. (2014) verified that gastrodin could significantly reduce Aβ deposition and glial cell activation in the brain of transgenic mice. These findings indicate that gastrodin has neuroprotective effects through anti-inflammatory and anti-amyloidosis effects. Nicotinamide has potential nerve protective effects (Rennie et al., 2015). Experiments have confirmed that the cognitive function of 3xTg AD mice could be improved after 8 months of treatment of nicotinamide; Aβ and hyperphosphorylated tau protein lesions in the hippocampus and the cerebral cortex were reduced. At the same time, nicotinamide can also reduce autophagosome accumulation by enhancing lysosome/autophagolysosome acidification, thereby protecting mitochondrial integrity (Liu et al., 2013). Umbelliferone could significantly increase the content of Nrf2 and HO-1 in rats, which have cognitive dysfunction induced by streptozotocin (Hindam et al., 2014). In summary, salidroside was not only showing high relevance through network pharmacology analysis but also could be absorbed *in vivo* and demonstrated its active effect in some references. Therefore, salidroside is one of the neuroprotective substances in Formula 9002A. In addition, gastrodin, niacinamide, and umbelliferon might also be potential active substances.

Furthermore, the pharmacokinetic properties of these compounds were studied. Due to the fact that limited time points of niacinamide and umbelliferon were detected, only salidroside and gastrodin were selected. The result showed that the half-life of salidroside in plasma was 2.117 h after the administration of Formula 9002A, which was nearly 2- to 3-folds higher than that 1.16 h by intravenous injection and 0.64 h by gavage of salidroside monomer (Naseri-Nosar and Ziora, 2018). A similar phenomenon was also found in the pharmacokinetic parameters of gastrodin. The half-life of gastrodin (2.579 h) after administration of Formula 9002A was almost 2-fold higher than that by intragastric or intravenous injection of gastrodin monomer, indicating that the compounds in compatibility with TCM either have a competitive effect on the absorption rate or have interaction leading to extended half-life and time of action. Another pharmacokinetic parameter of concern is the mean residence time (MRT), which describes the average length of time a drug stays in a living organism. The MRT of salidroside and gastrodin was calculated to be 2.905 and 3.567 h, respectively, both of which were larger than the MRT of the two compounds reported in the literatures, proving that compatibility of Chinese medicine did prolong the retention time of monomer drugs *in vivo* (Naseri-Nosar and Ziora). In fact, TCM formulations usually come from the records of classic books or according to the experience of pharmacists, but they also follow certain rules, that is, the rationality of compatibility. The most basic rationality of the compatibility of Chinese herbal compound is to increase the efficacy and reduce toxicity (Xia et al., 2019). The combination of two or more herbs may increase the exposure and absorption effect of active ingredients and prolong the retention time of drugs or reduce the absorption effect and exposure degree of toxic ingredients to relieve the burden of TCM metabolism.

In summary, pharmacodynamics results including cell experiments and animal behavioral experiments demonstrated that Formula 9002A exerted neuroprotective effect. Furthermore, the network pharmacology of Formula 9002A was investigated to clarify action mechanism and potential active substances, which showed that the ingredients including mandenol, turmeronol B, β-pinene, salidroside, rhodionin, isorhamnetin, beta-carotene, and biflavone and targets including ESR1, RELA, CYP17A1, APP, TNF, AR, and GSK3β showed high relevance with AD. Among them, salidroside and gastrodin could be absorbed *in vivo* and their half-times and MRTs both increased to exert the extended action time. Additionally, the main targets APP and GSK3β in the Alzheimer’s disease pathway were verified. The present study employed an integrated strategy to demonstrate the main active substances in Formula 9002A and potential targets of Formula 9002A in the treatment of AD, which provide confidence and scientific basis in the efficacy of Formula 9002A in the treatment of AD and give the new idea and method for the development of Chinese medical prescription for neurodegenerative diseases.

## Data Availability

The original contributions presented in the study are included in the article/Supplementary Material, further inquiries can be directed to the corresponding authors.
