# Supplementary material for: Pharmacodynamics, Network Pharmacology, and Pharmacokinetics of Chinese Medicine Formula 9002A in the Treatment of Alzheimer’s Disease
Source: Front Pharmacol. 2022 Apr 8;13:849994. doi: 10.3389/fphar.2022.849994 (PMC9026172; doi:10.3389/fphar.2022.849994)
Supplement: Supplementary file 1 [file Table1.DOCX]

Behavior test

1. Animal grouping and administration

18 ICR mice were randomly divided into 3 groups (control group, Aβ model group, treatment group), with 6 mice in each group. The mice in the control group and the Aβ model group were given physiological saline, and the mice in the treatment group were given intragastric administration of Formula 9002A at a dose of 3g/kg/day for 36 days.

2. Animals modeling

On the 21st day of feeding, the mice in the model group and the administration group were anesthetized by intraperitoneal injection of 5% chloral hydrate at a dose of 10 mg/kg according to the body weight of the mice. After about 6 minutes, it is observed whether the mouse has entered anesthesia. If the mice were deeply anesthetized, they were fixed on the stereotaxic device (RWD life science, Shenzhen, China), then removing the hair on the top of its head and moistening the mouse's eyes with eye drops. Furthermore, a serial of operations were applied, including taking the center of the mouse head as an incision to expose the bregma, and using a cotton swab dipped in a small amount of normal saline to wipe the surface of the skull to make the bregma more clearly. According to the mouse brain map combined with the brain area required for the experiment, the Aβ_1-42_ oligomer was slowly injected at a fixed coordinate (downward 3.1mm; side opening ±1.9mm; depth 2.0mm) with a micro syringe at a constant rate of 0.2μL/min (1μg/side), then stopping the needle for 5 minutes to prevent the backflow of the drug, slowly pulling out the micro syringe, suturing the wound with a sterile suture needle, and applying erythromycin to the wound to prevent postoperative infection. The control group was injected with an equal volume of saline in the same way. A behavioral test will be performed one week later.

3. Open field test

On the 27th day of feeding, the mice in each group were subjected to the OFT (Mobile Datum, Shanghai, China) in turn. The experiment requires keeping the environment dim and quiet. It is carried out in an open box with a square of 0.4×0.4m on the bottom and walls on all sides. The mouse was put with the back of the two objects into the open box and keeping the nose of the mouse at the same distance from the two objects. After putting the mouse in, the video equipment was immediately turned on and the number of upright times and threading times of the mouse in the open box for 3 minutes were recorded. After 3 minutes, the mice were returned to their original cages.

4. New object recognition test

When feeding to the 28th day (that is, one day after the open field experiment), the NOR (Mobile Datum, Shanghai, China) was performed. It is carried out in an open box with a square bottom surface of 0.4×0.4 m and walls on all sides. It is needed to prepare three stones, of which the No. 1 and No. 2 stones were exactly the same, and the No. 3 and No. 2 stones were different. The animals were placed in an open box to adapt for a period of time before the experiment. After 10 minutes, the two stones No. 1 and No. 2 were placed on the opposite ends of the open box. Then the mouse was put with the back of the two objects into the open box and keeping the nose of the mouse at the same distance from the two objects. After putting the mouse in, the video equipment was immediately turned on to record the mouse's contact with the two rocks, including the time when the mouse's nose or mouth touches the object. After 3 minutes, the mice were returned to their original cages. The retest was carried out at next day (the 29th day of feeding), and the No. 2 stone block in the field was replaced with the No. 3 stone block. The other steps were the same as the first day.

5. Morris water maze test

On the 31st day of feeding, the mice were subjected to a MWM (Mobile Datum, Shanghai, China) in sequence, which was divided into two parts: positioning navigation and a space exploration experiment. Positioning navigation test: The day before the test (the 30th day of feeding), the mice were put into the pool to swim freely for 90s to adapt to the environment. Before the test, the platform was placed in the center of the proximal quadrant and hidden 2 cm below the water surface. The mouse was placed into the pool along the middle of the 4 quadrants of the pool, then turning on the automatic camera system, and recording the time it takes for the mouse to find the platform (the escape latency period) and the swimming route. The longest escape latency is set to 90s. The camera system stopped recording after 90s. If the mouse found the platform within 90s and stayed on the platform for more than 3s, the timing was stopped and considered as the escape latency of the mouse. If the mouse did not find the platform within 90s, it was led to the platform and stayed for 20s, and the escape latency was recorded as 90s. This part of the experiment lasted for 4 days. The space exploration experiment was started on the 5th day (the 36th day of feeding). The mouse entered the water from the midpoint of the pool wall in the opposite quadrant of the platform, swimming freely for 90 seconds, and its swimming trajectory was recorded. After the video is over, the number of times the mice have crossed the platform was counted and the distance ratio of the mice in the target quadrant was calculated.
