# Supplementary material for: Pharmacodynamics, Network Pharmacology, and Pharmacokinetics of Chinese Medicine Formula 9002A in the Treatment of Alzheimer’s Disease
Source: Front Pharmacol. 2022 Apr 8;13:849994. doi: 10.3389/fphar.2022.849994 (PMC9026172; doi:10.3389/fphar.2022.849994)
Supplement: Supplementary file 2 [file Table2.DOCX]

Table S1 124 active compounds of Formula 9002A.

| Mol ID | Molecule Name | MW | OB (%) | BBB | DL |
| --- | --- | --- | --- | --- | --- |
| MOL000006 | luteolin | 286.25 | 36.16 | -0.84 | 0.25 |
| MOL000008 | apigenin | 270.25 | 23.06 | -0.61 | 0.21 |
| MOL000023 | [Hemo-sol](http://tcmspw.com/molecule.php?qn=23) | 136.26 | 39.84 | 2.12 | 0.02 |
| MOL000040 | scopoletin | 192.18 | 27.77 | 0.3 | 0.08 |
| MOL000090 | curcumin (enol form) | 368.41 | 5.15 | -0.76 | 0.41 |
| MOL000103 | benzoic acid | 138.13 | 30.15 | 0.21 | 0.03 |
| MOL000244 | [(+)-Borneol](http://tcmspw.com/molecule.php?qn=244) | 154.28 | 81.8 | 1.47 | 0.05 |
| MOL000263 | oleanolic acid | 456.78 | 29.02 | 0.07 | 0.76 |
| MOL000346 | Succinic acid | 118.1 | 29.62 | -0.71 | 0.01 |
| MOL000354 | isorhamnetin | 316.28 | 49.6 | -0.54 | 0.31 |
| MOL000358 | [beta-sitosterol](http://tcmspw.com/molecule.php?qn=358) | 414.79 | 36.91 | 0.99 | 0.75 |
| MOL000359 | [sitosterol](http://tcmspw.com/molecule.php?qn=359) | 414.79 | 36.91 | 0.87 | 0.75 |
| MOLl000415 | rutin | 610.57 | 3.2 | -2.75 | 0.68 |
| MOL000422 | kaempferol | 286.25 | 41.88 | -0.55 | 0.24 |
| MOL000449 | [Stigmasterol](http://tcmspw.com/molecule.php?qn=449) | 412.77 | 43.83 | 1 | 0.76 |
| MOL000475 | [anethole](http://tcmspw.com/molecule.php?qn=475) | 148.22 | 32.49 | 1.81 | 0.03 |
| MOL000485 | [(+)-alpha-Pinene](http://tcmspw.com/molecule.php?qn=485) | 136.26 | 46.25 | 2.18 | 0.05 |
| MOL000493 | [campesterol](http://tcmspw.com/molecule.php?qn=493) | 400.76 | 37.58 | 0.93 | 0.71 |
| MOL000511 | ursolic acid | 456.78 | 16.77 | 0.07 | 0.75 |
| MOL000513 | gallic acid | 170.13 | 31.69 | -0.54 | 0.04 |
| MOL000522 | arctiin | 534.61 | 34.45 | -1.5 | 0.84 |
| MOL000536 | matairesinoside | 520.58 | 24.88 | -1.9 | 0.84 |
| MOL000612 | [(-)-alpha-cedrene](http://tcmspw.com/molecule.php?qn=612) | 204.39 | 55.56 | 2.16 | 0.1 |
| MOL000635 | vanilin | 152.16 | 52 | 0.41 | 0.03 |
| MOL000771 | hydroxycinnamic acid | 164.17 | 43.29 | 0.13 | 0.04 |
| MOL000898 | (1S,6R,7R)-4-isopropylidene-1-methyl-7-(3-oxobutyl) norcaran-3-one | 234.37 | 34.17 | 0.61 | 0.11 |
| MOL000900 | [(5R,6R)-5-isopropenyl-3,6-dimethyl-6-vinyl-5,7-dihydrobenzofuran-4-one](http://tcmspw.com/molecule.php?qn=900) | 230.33 | 57.05 | 1.32 | 0.11 |
| MOL000901 | [BRN 3094585](http://tcmspw.com/molecule.php?qn=901) | 234.37 | 87.82 | 1.29 | 0.13 |
| MOL000902 | [curcumol](http://tcmspw.com/molecule.php?qn=902) | 236.39 | 103.55 | 1.23 | 0.13 |
| MOL000905 | [(+)-beta-Pinene](http://tcmspw.com/molecule.php?qn=905) | 136.26 | 44.77 | 2.29 | 0.05 |
| MOL000910 | [Germacron](http://tcmspw.com/molecule.php?qn=910) | 218.37 | 32.5 | 1.53 | 0.07 |
| MOL000917 | [cineole](http://tcmspw.com/molecule.php?qn=917) | 154.28 | 59.96 | 1.97 | 0.05 |
| MOL000944 | [(6R)-2-methyl-6-(4-methylphenyl) hept-2-en-4-one](http://tcmspw.com/molecule.php?qn=944) | 216.35 | 40.84 | 1.5 | 0.07 |
| MOL000945 | bisdemethoxycurcumin | 308.35 | 3.55 | -0.48 | 0.26 |
| MOL000947 | [(6Z,10S)-3-isopropylidene-6,10-dimethyl-cyclodec-6-ene-1,4-quinone](http://tcmspw.com/molecule.php?qn=947) | 234.37 | 35.77 | 0.77 | 0.08 |
| MOL000949 | [(3S,3aS,8aR)-3-hydroxy-5-isopropylidene-3-methyl-8-methylene-2,3a,4,8a-tetrahydro-1H-azulen-6-one](http://tcmspw.com/molecule.php?qn=949) | 234.37 | 46.11 | 0.55 | 0.1 |
| MOL000953 | Cholesterol | 386.73 | 37.87 | 1.13 | 0.68 |
| MOL000954 | [α-turmerone](http://tcmspw.com/molecule.php?qn=954) | 218.37 | 31.47 | 1.49 | 0.07 |
| MOL000955 | [turmeronol A](http://tcmspw.com/molecule.php?qn=955) | 232.35 | 59.42 | 0.84 | 0.08 |
| MOL000956 | [(6S)-2-methyl-6-[(1S)-4-methylene-1-cyclohex-2-enyl] hept-2-en-4-one](http://tcmspw.com/molecule.php?qn=956) | 218.37 | 43.91 | 1.63 | 0.07 |
| MOL000960 | [procurcumadiol](http://tcmspw.com/molecule.php?qn=960) | 250.37 | 69.82 | 0.42 | 0.13 |
| MOL000961 | (3S,3aS,8aR)-3-hydroxy-5-isopropylidene-3,8-dimethyl-2,3a,4,8a-tetrahydro-1H-azulen-6-one | 234.37 | 34.4 | 0.55 | 0.1 |
| MOL000963 | [bisacumol](http://tcmspw.com/molecule.php?qn=963) | 218.37 | 31.41 | 1.24 | 0.07 |
| MOL000966 | [turmeronol B](http://tcmspw.com/molecule.php?qn=966) | 232.35 | 35.84 | 1.12 | 0.08 |
| MOL000969 | [Dicumene](http://tcmspw.com/molecule.php?qn=969) | 238.4 | 38.08 | 2.04 | 0.11 |
| MOL000970 | [Curlone](http://tcmspw.com/molecule.php?qn=970) | 218.37 | 32.52 | 1.5 | 0.07 |
| MOL000972 | [germacrone-13-al](http://tcmspw.com/molecule.php?qn=972) | 232.35 | 42 | 0.73 | 0.09 |
| MOL001323 | Sitosterol alpha 1 | 426.8 | 43.28 | 0.97 | 0.78 |
| MOL001456 | Citric acid | 192.14 | 56.22 | -1.38 | 0.05 |
| MOL001490 | [bis[(2S)-2-ethylhexyl] benzene-1,2-dicarboxylate](http://tcmspw.com/molecule.php?qn=1490) | 390.62 | 43.59 | 0.68 | 0.35 |
| MOL001494 | [Mandenol](http://tcmspw.com/molecule.php?qn=1494) | 308.56 | 42 | 1.14 | 0.19 |
| MOL001495 | Ethyl linolenate | 306.54 | 46.1 | 1.12 | 0.2 |
| MOL001525 | Daucosterol | 414.79 | 36.91 | 1.15 | 0.75 |
| MOL001603 | demethoxycurcumin | 338.38 | 4.37 | -0.59 | 0.33 |
| MOL001843 | P-hydroxybenzaldehyde | 122.13 | 29.98 | 0.63 | 0.02 |
| MOL001979 | Lanosterol | 426.8 | 42.12 | 1.18 | 0.75 |
| MOL002225 | cinnamyl alcohol | 134.19 | 38.35 | 1.09 | 0.02 |
| MOL002320 | Sitosterol | 414.79 | 36.91 | 0.88 | 0.75 |
| MOL002372 | [Campesterol](http://tcmspw.com/molecule.php?qn=2372) | 410.8 | 33.55 | 1.77 | 0.42 |
| MOL002581 | curcumin (keto form) | 368.41 | 4.37 | -0.6 | 0.41 |
| MOL002737 | flavonoid | 286.25 | 18.97 | -0.54 | 0.24 |
| MOL002773 | [beta-carotene](http://tcmspw.com/molecule.php?qn=2773) | 536.96 | 37.18 | 1.52 | 0.58 |
| MOL002883 | [Ethyl oleate (NF)](http://tcmspw.com/molecule.php?qn=2883) | 310.58 | 32.4 | 1.1 | 0.19 |
| MOL002929 | salidroside | 300.34 | 7.01 | -1.41 | 0.02 |
| MOL002930 | tyrosol | 138.18 | 33.81 | 0.25 | 0.02 |
| MOL003578 | [Cycloartenol](http://tcmspw.com/molecule.php?qn=3578) | 426.8 | 38.69 | 1.33 | 0.78 |
| MOL004067 | Nootkatone | 218.37 | 33.04 | 1.51 | 0.1 |
| MOL004333 | [Ar-turmerone](http://tcmspw.com/molecule.php?qn=954) | 216.35 | 60.86 | 1.6 | 0.07 |
| MOL005043 | [campest-5-en-3beta-ol](http://tcmspw.com/molecule.php?qn=5043) | 400.76 | 37.58 | 0.94 | 0.71 |
| MOL005438 | [campesterol](http://tcmspw.com/molecule.php?qn=5438) | 400.76 | 37.58 | 0.95 | 0.71 |
| MOL005573 | genkwanin | 284.28 | 37.13 | -0.24 | 0.24 |
| MOL006209 | [cyanin](http://tcmspw.com/molecule.php?qn=6209) | 411.66 | 47.42 | 0.99 | 0.76 |
| MOL006838 | anthocyanin | 207.26 | 55.35 | 1.62 | 0.11 |
| MOL006927 | P-hydroxybenzyl alcohol | 124.15 | 55.19 | 0.34 | 0.02 |
| MOL007179 | [Linolenic acid ethyl ester](http://tcmspw.com/molecule.php?qn=7179) | 306.54 | 46.1 | 1.09 | 0.2 |
| MOL007449 | [24-methylidenelophenol](http://tcmspw.com/molecule.php?qn=7449) | 412.77 | 44.19 | 1 | 0.75 |
| MOL007986 | gastrodin | 286.31 | 8.19 | -2.29 | 0.17 |
| MOL008173 | Sitogluside | 414.79 | 36.91 | 0.85 | 0.75 |
| MOL009355 | [sitosterol palmitate](http://tcmspw.com/molecule.php?qn=9355) | 653.25 | 30.91 | 0.7 | 0.4 |
| MOL009356 | Tectochrysin | 268.28 | 9.57 | 0.27 | 0.2 |
| MOL009604 | [14b-pregnane](http://tcmspw.com/molecule.php?qn=9604) | 288.57 | 34.78 | 1.95 | 0.34 |
| MOL009612 | [(24R)-4alpha-Methyl-24-ethylcholesta-7,25-dien-3beta-ylacetate](http://tcmspw.com/molecule.php?qn=9612) | 482.87 | 46.36 | 1 | 0.84 |
| MOL009617 | [24-ethylcholest-22-enol](http://tcmspw.com/molecule.php?qn=9617) | 414.79 | 37.09 | 0.99 | 0.75 |
| MOL009618 | [24-ethylcholesta-5,22-dienol](http://tcmspw.com/molecule.php?qn=9618) | 412.77 | 43.83 | 0.84 | 0.76 |
| MOL009620 | [24-methyl-31-norlanost-9(11)-enol](http://tcmspw.com/molecule.php?qn=9620) | 428.82 | 38 | 0.93 | 0.75 |
| MOL009621 | [24-methylenelanost-8-enol](http://tcmspw.com/molecule.php?qn=9621) | 440.83 | 42.37 | 1.18 | 0.77 |
| MOL009622 | [Fucosterol](http://tcmspw.com/molecule.php?qn=9622) | 412.77 | 43.78 | 1.01 | 0.76 |
| MOL009631 | [31-Norcyclolaudenol](http://tcmspw.com/molecule.php?qn=9631) | 440.83 | 38.68 | 0.94 | 0.81 |
| MOL009633 | [31-norlanost-9(11)-enol](http://tcmspw.com/molecule.php?qn=9633) | 414.79 | 38.35 | 1.03 | 0.72 |
| MOL009634 | [31-norlanosterol](http://tcmspw.com/molecule.php?qn=9634) | 412.77 | 42.2 | 1.03 | 0.73 |
| MOL009635 | [4,24-methyllophenol](http://tcmspw.com/molecule.php?qn=9635) | 414.79 | 37.83 | 1.1 | 0.75 |
| MOL009639 | [Lophenol](http://tcmspw.com/molecule.php?qn=9639) | 400.76 | 38.13 | 1.1 | 0.71 |
| MOL009640 | [4alpha,14alpha,24-trimethylcholesta-8,24-dienol](http://tcmspw.com/molecule.php?qn=9640) | 426.8 | 38.91 | 1.06 | 0.76 |
| MOL009641 | [4alpha,24-dimethylcholesta-7,24-dienol](http://tcmspw.com/molecule.php?qn=9641) | 412.77 | 42.65 | 0.98 | 0.75 |
| MOL009642 | [4alpha-methyl-24-ethylcholesta-7,24-dienol](http://tcmspw.com/molecule.php?qn=9642) | 426.8 | 42.3 | 1.08 | 0.78 |
| MOL009644 | [6-Fluoroindole-7-Dehydrocholesterol](http://tcmspw.com/molecule.php?qn=9644) | 402.7 | 43.73 | 0.54 | 0.72 |
| MOL009650 | [Atropine](http://tcmspw.com/molecule.php?qn=9650) | 289.41 | 42.16 | 0.39 | 0.19 |
| MOL009653 | [Cycloeucalenol](http://tcmspw.com/molecule.php?qn=9653) | 426.8 | 39.73 | 1.04 | 0.79 |
| MOL009656 | [(E, E)-1-ethyl octadeca-3,13-dienoate](http://tcmspw.com/molecule.php?qn=9656) | 308.56 | 42 | 1.11 | 0.19 |
| MOL009677 | [lanost-8-en-3beta-ol](http://tcmspw.com/molecule.php?qn=9677) | 428.82 | 34.23 | 1.25 | 0.74 |
| MOL009678 | [lanost-8-enol](http://tcmspw.com/molecule.php?qn=9678) | 428.82 | 34.23 | 1.12 | 0.74 |
| MOL009681 | [Obtusifoliol](http://tcmspw.com/molecule.php?qn=9681) | 426.8 | 42.55 | 1.25 | 0.76 |
| MOL010234 | [delta-Carotene](http://tcmspw.com/molecule.php?qn=10234) | 536.96 | 31.8 | 1.42 | 0.55 |
| MOL010246 | flavonol | 238.25 | 47.91 | 0.77 | 0.16 |
| MOL011051 | ginkgolic acid | 346.56 | 20.18 | 0.61 | 0.32 |
| MOL011060 | Ginkgolide A | 408.44 | 13.82 | -0.5 | 0.74 |
| MOL011061 | Ginkgolide B | 424.44 | 46.14 | -0.73 | 0.73 |
| MOL011578 | bilobalide | 326.33 | 84.42 | -1.34 | 0.36 |
| MOL011587 | Ginkgolide C | 440.44 | 48.33 | -1.76 | 0.73 |
| MOL011588 | Ginkgolide J | 424.44 | 44.84 | -1.59 | 0.74 |
| MOL011643 | pectin | 194.16 | 40.39 | -3.12 | 0.06 |
|  | 2-O-b-D-glucopyranosyl-L-ascorbic acid | 338.27 |  |  |  |
|  | biflavone | 442.5 |  |  |  |
|  | yakuchinone A | 312.4 |  |  |  |
|  | calendic acid | 278.4 |  |  |  |
|  | vanilyl alcohol | 153.05 |  |  |  |
|  | parishin C | 728.6 |  |  |  |
|  | parishin A | 996.9 |  |  |  |
|  | parishin B | 728.6 |  |  |  |
|  | parishin E | 460.4 |  |  |  |
|  | bisacurone B | 252.35 |  |  |  |
|  | rosarin | 428.4 |  |  |  |
|  | rosavin | 428.4 |  |  |  |
|  | rhodionin | 448.4 |  |  |  |
