# Supplementary material for: Pharmacodynamics, Network Pharmacology, and Pharmacokinetics of Chinese Medicine Formula 9002A in the Treatment of Alzheimer’s Disease
Source: Front Pharmacol. 2022 Apr 8;13:849994. doi: 10.3389/fphar.2022.849994 (PMC9026172; doi:10.3389/fphar.2022.849994)
Supplement: Supplementary file 3 [file Table3.DOCX]

Table S2 101 genes matched with AD-related targets in the database.

| Gene Name | | | | | | |
| --- | --- | --- | --- | --- | --- | --- |
| ESR1 | ABCG2 | ADRA2A | MAOB | HDAC3 | NR3C1 | DPP4 |
| PTGS2 | CYP1A2 | ADRA2C | CHRM1 | MMP9 | PNMT | ACHE |
| AR | MAPK10 | CNR1 | CHRNA4 | HDAC2 | CNR2 | PTPRC |
| RELA | AHR | ADRA2B | ABCB1 | HDAC4 | CXCR3 | SIRT1 |
| APP | MCL1 | S1PR2 | DNMT1 | HDAC6 | CHRM2 | ACE |
| ESR2 | CYP17A1 | MAOA | TERT | ALOX5 | S1PR5 | MME |
| CYP19A1 | NOS3 | CASP9 | HDAC1 | HNF4A | MGLL | ITGA2B |
| NOS2 | HSP90AA1 | PLA2G1B | RARB | DRD5 | CASR | S1PR3 |
| CSNK2A1 | CYP2C9 | SLC6A4 | THRA | GRM2 | SRD5A2 | TBXA2R |
| PTPN1 | CYP2C19 | CA9 | ICAM1 | GRIA2 | PPARG | ADORA1 |
| PTGFR | MMP2 | BCL2A1 | RARA | CHRNA7 | TLR9 | OPRD1 |
| PTGS1 | CDC42 | PTGES | RXRA | SLC6A2 | PPARA | S1PR1 |
| TNF | FAAH | RAC1 | SIRT2 | CASP3 | CTSB | GRIN2B |
| GSK3β | MMP1 | HTR5A | DRD1 | HTR2A | CES1 | OPRM1 |
| GRM4 | PIM1 | TOP1 |  |  |  |  |
