# Supplementary material for: Pharmacodynamics, Network Pharmacology, and Pharmacokinetics of Chinese Medicine Formula 9002A in the Treatment of Alzheimer’s Disease
Source: Front Pharmacol. 2022 Apr 8;13:849994. doi: 10.3389/fphar.2022.849994 (PMC9026172; doi:10.3389/fphar.2022.849994)
Supplement: Supplementary file 4 [file Table4.DOCX]

Table S4 Abbreviations of 94 active compounds of Formula 9002A in C-T-P network.

| Abbreviation | Mol ID | Molecule Name |
| --- | --- | --- |
| GQ1 | MOL009604 | [14b-pregnane](http://tcmspw.com/molecule.php?qn=9604) |
| GQ2 | MOL009681 | [24-ethylcholesta-5,22-dienol](http://tcmspw.com/molecule.php?qn=9618) |
| GQ3 | MOL009677 | [lanost-8-en-3beta-ol](http://tcmspw.com/molecule.php?qn=9677) |
| GQ4 | MOL009621 | [24-methylenelanost-8-enol](http://tcmspw.com/molecule.php?qn=9621) |
| GQ5 | MOL001979 | Lanosterol |
| GQ7 | MOL001495 | Ethyl linolenate |
| GQ8 | MOL009678 | [lanost-8-enol](http://tcmspw.com/molecule.php?qn=9678) |
| GQ9 | MOL009656 | [(E, E)-1-ethyl octadeca-3,13-dienoate](http://tcmspw.com/molecule.php?qn=9656) |
| GQ10 | MOL009634 | [31-norlanosterol](http://tcmspw.com/molecule.php?qn=9634) |
| GQ11 | MOL009622 | [Fucosterol](http://tcmspw.com/molecule.php?qn=9622) |
| GQ12 | MOL007449 | [24-methylidenelophenol](http://tcmspw.com/molecule.php?qn=7449) |
| GQ13 | MOL009617 | [24-ethylcholest-22-enol](http://tcmspw.com/molecule.php?qn=9617) |
| GQ15 | MOL001323 | Sitosterol alpha 1 |
| GQ16 | MOL005438 | [campesterol](http://tcmspw.com/molecule.php?qn=5438) |
| GQ17 | MOL009618 | [24-ethylcholesta-5,22-dienol](http://tcmspw.com/molecule.php?qn=9618) |
| GQ18 | MOL009650 | [Atropine](http://tcmspw.com/molecule.php?qn=9650) |
| GQ19 | MOL000040 | scopoletin |
| GQ20 | MOL007449 | [24-methylidenelophenol](http://tcmspw.com/molecule.php?qn=7449) |
| GQ21 | MOL009617 | [24-ethylcholest-22-enol](http://tcmspw.com/molecule.php?qn=9617) |
| HJT1 | MOL002930 | tyrosol |
| HJT2 | MOL002225 | cinnamyl alcohol |
| HJT3 | MOL002929 | salidroside |
| HJT4 |  | Rhodionin |
| HJT5 |  | Rhodiosin |
| JH1 | MOL000023 | [Hemo-sol](http://tcmspw.com/molecule.php?qn=23) |
| JH2 | MOL000244 | [(+)-Borneol](http://tcmspw.com/molecule.php?qn=244) |
| JH3 | MOL000475 | [anethole](http://tcmspw.com/molecule.php?qn=475) |
| JH4 | MOL000485 | [(+)-alpha-Pinene](http://tcmspw.com/molecule.php?qn=485) |
| JH5 | MOL000493 | [campesterol](http://tcmspw.com/molecule.php?qn=493) |
| JH6 | MOL000612 | [(-)-alpha-cedrene](http://tcmspw.com/molecule.php?qn=612) |
| JH7 | MOL000898 | isopropylidene-1-methyl-7-(3-oxobutyl) norcaran-3-one |
| JH8 | MOL000900 | 3,6-dimethyl-6-vinyl-5,7-dihydrobenzofuran-4-one |
| JH9 | MOL000905 | [(+)-beta-Pinene](http://tcmspw.com/molecule.php?qn=905) |
| JH10 | MOL000910 | [Germacron](http://tcmspw.com/molecule.php?qn=910) |
| JH11 | MOL000917 | [cineole](http://tcmspw.com/molecule.php?qn=917) |
| JH12 | MOL000944 | [(6R)-2-methyl-6-(4-methylphenyl) hept-2-en-4-one](http://tcmspw.com/molecule.php?qn=944) |
| JH14 | MOL000949 | methyl-8-methylene-2,3a,4,8a-tetrahydro-1H-azulen-6-one |
| JH16 | MOL000954 | [α-turmerone](http://tcmspw.com/molecule.php?qn=954) |
| JH17 | MOL000955 | [turmeronol A](http://tcmspw.com/molecule.php?qn=955) |
| JH18 | MOL000960 | [procurcumadiol](http://tcmspw.com/molecule.php?qn=960) |
| JH19 | MOL000961 | (3S,3aS,8aR)-3-hydroxy-5-isopropylidene-3,8-dimethyl-2,3a,4,8a-tetrahydro-1H-azulen-6-one |
| JH20 | MOL000963 | [bisacumol](http://tcmspw.com/molecule.php?qn=963) |
| JH21 | MOL000966 | [turmeronol B](http://tcmspw.com/molecule.php?qn=966) |
| JH22 | MOL000969 | [Dicumene](http://tcmspw.com/molecule.php?qn=969) |
| JH23 | MOL000972 | [germacrone-13-al](http://tcmspw.com/molecule.php?qn=972) |
| JH24 | MOL002581 | curcumin (keto form) |
| JH25 | MOL000090 | curcumin (enol form) |
| JH26 | MOL001603 | demethoxycurcumin |
| JH27 | MOL000945 | bisdemethoxycurcumin |
| JH28 | MOL004333 | [Ar-turmerone](http://tcmspw.com/molecule.php?qn=954) |
| SS1 | MOL002372 | [Campesterol](http://tcmspw.com/molecule.php?qn=2372) |
| SS2 | MOL002773 | β-carotene |
| SS3 | MOL006838 | anthocyanin |
| SS4 | MOL010246 | flavonol |
| SS6 | MOL000103 | benzoic acid |
| SS7 | MOL000771 | hydroxycinnamic acid |
| SS8 | MOL000415 | rutin |
| SS9 | MOL011643 | pectin |
| SLZ1 | MOL000511 | ursolic acid |
| SLZ2 | MOL000263 | oleanolic acid |
| SLZ3 | MOL000513 | gallic acid |
| TM1 | MOL002320 | Sitosterol |
| TM2 | MOL000635 | vanilin |
| TM3 |  | vanilyl alcohol |
| TM4 | MOL001843 | *p*-hydroxybenzaldehyde |
| TM5 | MOL006927 | *p*-hydroxybenzyl alcohol |
| TM6 | MOL000346 | Succinic acid |
| TM7 | MOL001456 | Citric acid |
| YZ1 | MOL000359 | [sitosterol](http://tcmspw.com/molecule.php?qn=359) |
| YZ2 | MOL004067 | Nootkatone |
| YZ3 | MOL009356 | Tectochrysin |
| YZ4 |  | Yakuchinone A |
| YX1 | MOL001490 | [bis[(2S)-2-ethylhexyl] benzene-1,2-dicarboxylate](http://tcmspw.com/molecule.php?qn=1490) |
| YX4 | MOL007179 | [Linolenic acid ethyl ester](http://tcmspw.com/molecule.php?qn=7179) |
| YX5 | MOL002883 | [Ethyl oleate (NF)](http://tcmspw.com/molecule.php?qn=2883) |
| YX6 | MOL005043 | [campest-5-en-3beta-ol](http://tcmspw.com/molecule.php?qn=5043) |
| YX7 |  | quercetin 3-O-[6-O-(α-L-rhamnosyl)- β-D-glucoside] |
| YX8 |  | quercetin 3-O-β-D-glucoside |
| YX9 |  | quercetin 3-O-[4-O-(α-L-rhamnosyl)-β-D-glucoside] |
| YX10 |  | quercetin 3-O-α-L-rhamnoside |
| YX11 |  | ginkgoneolic acid |
| YX12 |  | biflavone |
| YX13 | MOL011578 | bilobalide |
| YX14 | MOL011061 | Ginkgolide B |
| YX15 | MOL011060 | Ginkgolide A |
| YX16 | MOL000006 | luteolin |
| YX17 | MOL000008 | apigenin |
| YX18 | MOL000354 | isorhamnetin |
| YX19 | MOL005573 | genkwanin |
| YX20 | MOL011051 | ginkgolic acid |
| A | MOL001494 | [Mandenol](http://tcmspw.com/molecule.php?qn=1494) |
| B | MOL000953 | Cholesterol |
| C | MOL000358 | [beta-sitosterol](http://tcmspw.com/molecule.php?qn=358) |
| D | MOL002737 | flavonoid |
